# Evolution and homoplasy at the *Bem6* microsatellite locus in three sweetpotato whitefly (*Bemisia tabaci*) cryptic species

**DOI:** 10.1186/1756-0500-6-249

**Published:** 2013-07-02

**Authors:** Aaron M Dickey, Paula M Hall, Robert G Shatters, Cindy L Mckenzie

**Affiliations:** 1 USDA-ARS, U.S. Horticultural Research Laboratory, 2001 South Rock Rd, Fort Pierce, FL 34945, USA; 2 Mid-Florida Research & Education Center, University of Florida, 2725 Binion Rd, Apopka, FL 32703, USA; 3 Current address: Mid-Florida Research & Education Center, University of Florida, 2725 Binion Rd, Apopka, FL 32703, USA

**Keywords:** Tandem repeat, Homoplasy, Compound microsatellite, Stepwise mutation, *Bemisia tabaci*

## Abstract

**Background:**

The evolution of individual microsatellite loci is often complex and homoplasy is common but often goes undetected. Sequencing alleles at a microsatellite locus can provide a more complete picture of the common evolutionary mechanisms occurring at that locus and can reveal cases of homoplasy. Within species homoplasy can lead to an underestimate of differentiation among populations and among species homoplasy can produce a misleading interpretation regarding shared alleles and hybridization. This is especially problematic with cryptic species.

**Results:**

By sequencing alleles from three cryptic species of the sweetpotato whitefly (*Bemisia tabaci*), designated MEAM1, MED, and NW, the evolution of the putatively dinucleotide *Bem6* (CA_8_)_imp_ microsatellite locus is inferred as one of primarily stepwise mutation occurring at four distinct heptaucleotide tandem repeats. In two of the species this pattern yields a compound tandem repeat. Homoplasy was detected both among species and within species.

**Conclusions:**

In the absence of sequencing, size homoplasious alleles at the *Bem6* locus lead to an overestimate of alleles shared and hybridization among cryptic species of *Bemisia tabaci*. Furthermore, the compound heptanucleotide motif structure of a putative dinucleotide microsatellite has implications for the nomenclature of heptanucleotide tandem repeats with step-wise evolution.

## Background

Satellite DNA was originally described as the bands produced by genomic DNA in a CsCl buoyant density gradient that fell outside the principal band [1]. These bands were found to be common in eukaryotes, have a GC content different from the principal band (bulk single copy DNA), and consist of tandemly repeated sequences [2]. Tandem sequence repetition has since become synonomous with the term satellite DNA [3]. Tandemly repetitive sequences have two parameters, the sequence motif, and the copy number (e.g. ATTATTATT contains three copies of the ATT trinucleotide motif). The diversity/variability within each parameter has lead to the creation of multiple classification schemes [3-7] and synonyms [8-12] within tandem-repeat nomenclature, particularly when copy number is less than 10^3^.

Tandem repeat marker loci contain the repeat region together with conserved flanking sequence and are usually non-coding. Evolution inside the repeat region is relatively fast due to a mutation rate that is 10^3^-10^5^ times higher than that of the genome as a whole [12-14]. Evolution of the tandem repeats occurs primarily by DNA slippage during replication (microsatellites) [15,16] or by gene conversion and crossover during meiosis (minisatellites) [17] but see Richard and Paques [18]. In the majority of cases, slippage causes an allele to change size by one repeat motif at a time in a stepwise fashion while gene conversion can cause copy number changes in larger multiples [18]. Tandem repeats tend to mutate faster with increasing copy number [19,20], and tend to expand when copy number is low and contract when copy number is high [21-23]. This observation is consistent with upper size constraints on copy number [24]. Mutations can also occur in the area flanking a tandem repeat [25], or into the tandem repeat itself, thereby causing an imperfect or interrupted repeat [26]. These can cause size homoplasy; cases where alleles among individuals have the same-size fragments (identical in character state), but arose in different lineages and are thus not identical by descent.

*Bemisia tabaci* Gennadius is a cryptic species complex composed of at least 24 morphologically identical species [27,28]. Most of these species are regionally endemic, but two are globally invasive agricultural pests, infesting and feeding on hundreds of crop plants in many diverse agro-ecosystems [29]. These species originated in the region bordering the Mediterranean Basin (MED) and in the Middle East/Asia Minor (MEAM1) region, and have also been referred to extensively in the literature as biotype Q and biotype B, respectively [28]. The recent arrival of MED to the United States in 2004 [30] raised concerns over possible hybridization with MEAM1, established in the United States since the mid 1980’s [31]. This outcome at present seems an unlikely prospect given that MEAM1 and MED are almost completely reproductively isolated [32]. Laboratory hybrids can occur, but are both rare and sterile [33], while field hybrids are also rare and do not persist [34]. Monitoring the spread of MED within the U.S. and distinguishing molecularly among MED and MEAM1 has been a principal aim of the biotype Q taskforce [35]. During this effort, a partial sequence of the mtCO1 gene [36,37] has been the gold standard for molecular identification, but two microsatellite loci, *Bem6* and *Bem23*[38] have also been used as diagnostic markers [31,34]. Because of the importance of the *Bem6* nuclear locus in *B. tabaci* cryptic species determination in North America, its diversity among economically important species and the origin of shared alleles is of interest.

The *Bem6* microsatellite was described as an imperfect (CA_8_)_imp_ tandem repeat where CA is the dinucleotide motif and 8 is the copy number [38] (Table 1). However, it has been noted that among U.S. samples, alleles at this locus occur in multiples of 7 base pairs rather than the expected 2 base pair multiples [31]. In addition, apparent hybrids and shared alleles appeared to be present among MED, MEAM1, and endemic New World (NW) whitefly samples in the dataset [31,34]. Because of this, alleles at this locus were sequenced in order to 1) better characterize the tandem repeat nature of this marker, and 2) evaluate the possibility of hybrids and shared alleles at this locus among cryptic species.

**Table 1:** **Nucleotide sequence of the Bem6 (CA)**_**8 **_**imperfect microsatellite and flanking region**[38]

**Position**	**DNA Sequence**																																
1	T	T	A	C	A	C	T	T	A	A	C	A	C	C	A	G	A	A	C	T	T	T	T	A	A	T	C	A	T	A	C	A	A
34	A	T	C	A	T	T	T	T	C	C	T	A	A	**C**	**A**	**C**	**A**	**C**	**A**	***A***	***C***	***T***	***T***	**C**	**A**	**C**	**A**	**C**	**A**	***A***	**C**	**A**	**C**
67	**A**	C	T	A	T	A	C	A	A	A	T	T	C	A	T	A	T	C	A	A	C	C	A	T	T	A	C	A	A	C	A	C	A
100	C	C	T	T	T	T	C	A	T	T	A	G	T	A	T	A	G	C	A	T	T	A	C	T	C	C	T	A	A	C	A	C	A
133	A	G	T	T	C	A	T	A	A	T	T	A	G	T	A	T	T	A	T	A	A	C	A	T	A	A	G	C	C	A	T	C	

The repeat region is in bold and imperfections in the CA motif are italicized.

## Methods

**Figure 1:**
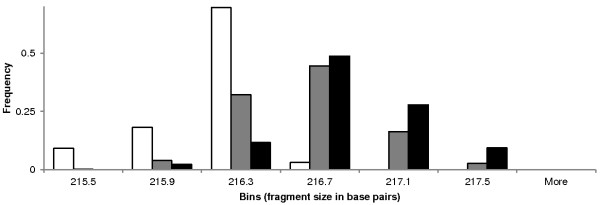
**Frequency histogram of allele size estimates calculated by Genemapper 4.0 based on the Rox400 standard curve for the alleles designated 216 NW (white, n = 33 fragments, median = 216.1 base pairs), 216 MEAM1 (gray, n = 4304 fragments, median = 216.4 base pairs), and 217 MED (black, n = 43 fragments, median = 216.6 base pairs).** Putatively shared alleles are present among the three cryptic species.

Between 2006 and 2011, 63 *Bem6* fragments representing 11 genotyped alleles were sequenced from 60 whiteflies (Table 2) as a supplementary part of large state and continent-wide surveys of *Bemisia tabaci* whiteflies [31,34]. The sequencing effort included samples from lab colonies as well as samples from Spain, Columbia, Israel, and Morocco. This represented between 1-2% of the whiteflies genotyped at this locus. Alleles not sequenced were very rare. Allele names are based on the average of estimated microsatellite fragment lengths calculated by Genemapper 4.0 (Applied Biosystems, Foster City, CA) rounded to the nearest base pair (Figure 1). These estimates are based on genotyping and form the basis for potential homoplasy, as alleles are rarely sequenced in practice [39]. The actual allele size as determined via sequencing may be different. Individuals were identified to species using MEAM1, MED, and NW specific primers based on the mitochondrial CO1 gene [36] with reference to the species-level groups identified by Dinsdale et al. [28].

**Table 2:** **Alleles sequenced from *B. tabaci *cryptic species**

**Estimated allele**	**Species**	**Individuals**	**Allele**
**size**		**sequenced**	frequency*
182	?	1	‡
189	NW	3	‡‡
195	NW	1	0.07
196	MED	3	<0.01
202	NW	2	0.51
203	MED	4	0.07
209	NW	2	0.16
210	MED	20	0.91
210	MEAM1	2	<0.01
216	NW	2	0.24
216	MEAM1	17	0.96
217	MED	3	0.01
223	MEAM1	3	0.04

63 microsatellite fragments sequenced from 60 individual whiteflies (some heterozygotes). Estimated allele size is rounded to the nearest whole number but see Figure 1.

*Estimates of North American field/greenhouse collected *Bemisia tabaci Bem6* allele frequencies by cryptic species taken from McKenzie et al. 2012.

‡ Singleton allele, species undetermined.

‡‡ Allele unique to lab colony.

Alleles were PCR amplified using the unlabeled primers described by De Barro et al. [38]. Amplified alleles were direct sequenced in most cases but were also cloned using an Invitrogen TOPO TA® Cloning Kit (Life Technologies, Carlsbad, CA) when it was necessary to sequence both alleles from a heterozygote. Sequencing reactions were run on an Applied Biosystems 3730XL DNA analyzer using a BigDye® Terminator v3.1 Cycle Sequencing Kit (Applied Biosystems). To be certain both alleles were sequenced from heterozygotes, a minimum of 16 colonies were sampled. Sequences were aligned first using the large gap setting in Sequencher 4.7 (Genecodes, Ann Arbor, MI) and then manually in Sequencher 4.7 and in Mesquite 2.74 [40].

## Results

### Homoplasy among whitefly species

**Figure 2:**
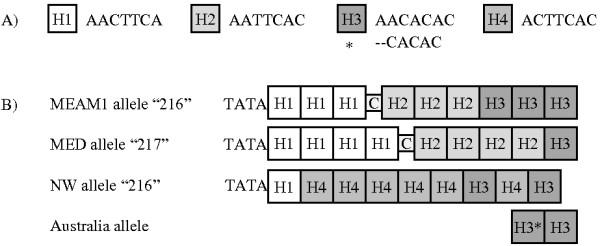
**Description and arrangement of heptanucleotide building blocks in a homoplasic Bem6 allele from four cryptic *B. tabaci *species. A)** Four different tandem repeat motifs revealed by sequencing Bem6 alleles. Species specific deviants are noted with asterisks. **B)** Homoplasy among species caused by differential presence/absence and order of motifs. An extra cytosine residue appears on MEAM1 and MED but not NW samples. Allele names are based on the average of estimated microsatellite fragment lengths calculated by Genemapper 4.0. The corresponding region of the originally described microsatellite [38] for the Australia species is shown for comparison. Four nucleotides absent from the originally described microsatellite precede the repeat region in MEAM1, MED, and NW whiteflies.

**Figure 3:**
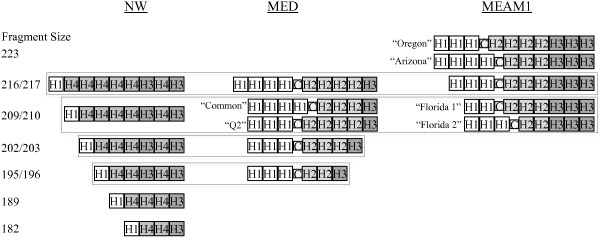
**Evolution via tandem heptanucleotide repeat insertion/deletion in three cryptic *B. tabaci *species; NW, MED, and MEAM1.** Within each species, only a single insertion or deletion is required to change from one allele to a progressively longer or shorter allele. Putative cases of among-species homoplasy are delimited with dotted line boxes. Cases of within-species homoplasy are indicated by allele labels in quotes. Descriptions of heptanucleotide building blocks are given in Figure 2.

In the three species, four perfect heptanucleotide tandem repeats designated H1-H4 (Figure 2a) were found preceding the 3′ flanking region of the *Bem6* microsatellite described by DeBarro et al. [38]. From 63 fragments representing 11 genotyped alleles (based on estimated size), 16 unique alleles (based on sequencing) were found (Figure 3). The sequence of the MEAM1 allele 216 was identical in 17 individual whiteflies collected from five U.S. States and contained a compound heptanucleotide repeat of motifs H1-H3 (Figure 2). The sequence of the NW allele 216 was identical in two whiteflies, one each from Texas and Mexico and is characterized by the presence of the H4 motif (Figure 2). The sequence of the MED allele 217 was identical in two whiteflies from Florida and one from Spain and contained a compound heptanucleotide repeat of motifs H1 and H2 (Figure 2). Instances of potential homoplasy among MEAM1 allele 216, NW allele 216, and MED allele 217 were caused by an overlapping distribution of allele size estimates (Figure 1). Additional cases of potential homoplasy (≤1 base pair estimated length difference) among cryptic species were found at alleles 209/210 and 195/196 (Figure 3). In MED whiteflies, allele 210 is the most common in North America with a frequency of 91%. Only two MEAM1 individuals had this allele meaning there would be a very low incidence (~1 in 2500) of MEAM1 individuals incorrectly identified. As reported previously [34], there was no evidence of allele sharing or hybridization among cryptic species at the *Bem6* locus (Figure 3).

### Homoplasy within whitefly species

Within species homoplasy was found for MED at allele 210 (Figure 3) and for MEAM1 at alleles 210 and 223 (Figure 3). In MED, two different 210 alleles were sequenced from 20 individuals from four countries and six U.S. States. The alleles differ from MED allele 217 by a single deletion or from MED allele 203 by a single insertion. This was the most frequent case of intraspecific homoplasy detected in the data. The allele designated “Q2” in Figure 3 was found in seven individuals collected in Guatemala and the U.S. States of Georgia and Oregon. The mitochondrial haplotypes of all seven individuals clustered with the haplotype Q2 described in McKenzie et al. [31] indicating maternal ancestry in the western Mediterranean region [41-43] (data not shown). The second allele 210 in MED was found in 13 individuals collected from Israel, Morocco, and the U.S. states of California, Pennsylvania, Florida, and Michigan. Of these 13, 11 had mitochondrial haplotypes that clustered with the haplotype Q1 described in McKenzie et al. [31] indicating maternal ancestry in the eastern Mediterranean region [41-43]. The other two individuals, a whitefly from Morocco and a whitefly from a California lab colony founded from individuals collected in Spain, had mitochondrial haplotypes consistent with western Mediterranean maternal ancestry (data not shown).

Two different MEAM1 210 alleles were sequenced, each from a single MEAM1 individual. Both alleles differ by a single heptanucleotide deletion from the common allele 216. Two different alleles with an estimated size of 223 base pairs were sequenced from three MEAM1 individuals. Both differ by a single heptanucleotide insertion from the common allele 216. The allele labeled “Arizona” in Figure 3 was sequenced from two individuals collected in Arizona while the other allele 223 was sequenced from an individual collected in Oregon. Allele 223 is only common in MEAM1 individuals from Arizona and New York greenhouse populations [43].

### Tandem repeat evolution

*Bem6* in all three cryptic species of whiteflies appears to evolve via insertions and/or deletions (indels) of four different heptanucleotide motifs and that these are generally repeated in tandem (Figure 3). Within each species, only a single indel is required to change from one allele to a progressively longer or shorter allele suggesting stepwise mutation occurred in the tandem repeat region within each cryptic species. The number of copies of heptanucleotide H3 is constant among individuals from both invasive species, MED (1 copy) and MEAM1 (3 copies), with evolution inferred as indels of the heptanucleotides designated H1 and H2 (Figure 3). Within NW, evolution is best explained by tandem indels of the heptanucleotide designated H4 (Figure 3). However, a non-tandem indel of H3 separates North American NW (alleles 195 and higher) and Columbian NW (allele 189). This is consistent with the divergent phylogenetic placement of North American and Columbian mtCO1sequences [44,45]. A single male individual collected in Florida carrying the allele 182 could not be identified to species due to insufficient sequence length in the mitochondrial barcode, but the sequence at the *Bem6* locus is most similar to other NW alleles. *Bem6* alleles in MEAM1 and MED contain compound tandem repeats of H1-H3 while NW features only H4 in tandem (Figure 3).

Within each species, the H3 motif appears to be the most stable, never showing copy number variation in tandem. This motif, AACACAC, is the most similar to the (CA_8_)_imp_ originally described by De Barro et al. [38]. Singleton copies of this motif are also found in both flanking regions of the originally described microsatellite (residues 45-51 and 94-100 in Table 1) and these persist in all three species studied here. The flanking region itself also appears relatively stable though a few species specific polymorphisms are present (Table 3).

**Table 3:** **Species specific polymorphisms in flanking regions of the *Bem6 *microsatellite**

	**Position**				
Species	22	23	43	69	129
MEAM1 + MED	**A**	**A**	**C**	**A**	**T**
Australia	**T**	**T**	**.**	**T**	**C**
NW	**T**	**.**	**A**	**.**	**T**

Dots denote identity with MEAM1 and MED alleles.

## Discussion

Generally the design of new microsatellites for each species separately is recommended [39], but this is not always possible when cryptic species are present or species level taxonomy is in flux. It should be noted that the *Bem6* microsatellite was originally isolated from the Australia species (P. De Barro, personal communication) see Dinsdale et al. [28] so it would be interesting to see if a similar pattern of heptanucleotide tandem repeat evolution is also present in this cryptic species. Determining the ancestral state of the Bem 6 locus within each cryptic species is not possible due to low allele number and paucity of home range sampling. Columbian NW are apparently ancestral to North and Central American NW [44] so allele 189 may be the ancestral state for NW. This should be investigated further with increased sampling throughout the home range of NW. It is not possible to speculate about the ancestral state of the *Bem6* locus within MED and MEAM1 due to the small number of alleles sampled in the native range. Further exploration of MED diversity in particular, including its apparently ancestral sub Saharan sub group [42], could help resolve this.

### Heptanucleotide repeats

Heptanucleotide tandem repeats have not received a lot of attention and this could be due, in part, to their exclusion from some classical definitions of both microsatellites and minisatellites e.g. [3]. While many accept some general rules for classifying tandemly repetitive DNA sequences [16,39], several authors have argued that some of these rules may be arbitrary [7,46]. Heptanucleotide repeats have been shown to be more common than tetra- and penta-nucleotide repeats in several plant taxa [47] suggesting that defining microsatellites as motifs between 1 and 6 nucleotides in length may also be unnecessarily exclusive. The data presented here is consistent with step-wise evolution, indicating that the four heptanucleotide motifs probably evolve like microsatellites [48] rather than minisatellites [17]. In addition, the alleles described may also have arisen via length independent slippage [49,50] expected when copy number is low.

### Compound microsatellites

For the species in this study, a microsatellite originally described as an imperfect dinucleotide repeat is characterized as a series of perfect heptanucleotide repeats. This change seems to be a function of the particular species in question as the motifs described here never occur in tandem in the original microsatellite (Figure 2). ‘Imperfections’ or ‘interruptions’ are commonly invoked in the tandem repeat literature [16,51,52] and are characterized this way because they break up a long perfect microsatellite into two shorter microsatellites [53]. But an interruption can also lead to the creation of a new ‘proto’ microsatellite [54,55]. If the newly created sequence motif is duplicated adjacent to the original repeat, it will form a compound microsatellite. This may be a common occurrence as compound microsatellites are 15 times more abundant in genomic DNA than random microsatellite distribution expectations [55].

**Figure 4:**
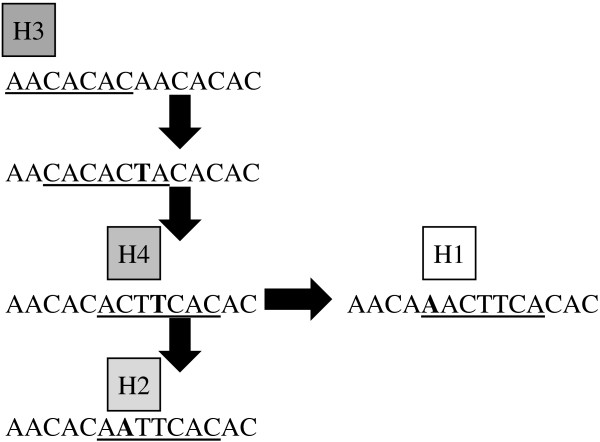
**Possible nucleotide substitution pathway separating the H1, H2, and H4 motifs from a doublet of the H3 motif.** Substitutions are in bold. The H3 heptanucleotide is present in all four cryptic whitefly species, the putative intermediate motif is only present in the Australia species.

To our knowledge, this is the first report of a compound heptanucleotide tandem repeat. While the pattern both within and among species is consistent with stepwise evolution of tandem repeats, choosing among alternative alignments was difficult and probably exacerbated by the absence of guanine in the repeat area. In the end, the alignment selected was the only one found where withinspecies size-sequential alleles were only separated by one mutation step. The next best alignment also featured four different heptanucleotide motifs, but had two additional point mutations separating NW alleles 189 and 195. One possible mutation pathway among the four identified motifs is given in Figure 4 with either one or two point mutations separating each motif. Kofler et al. [55] found that the motifs comprising most compound microsatellite pairs differed by a single mutation and suggested that such mutations represent the dominant mechanism underlying the origin of compound microsatellites. The evolutionary fate of a given microsatellite ‘interruption’ will probably depend on the structural properties of the new motif [56], but may ultimately yield a compound microsatellite.

In the evolution via nucleotide substitution scenario shown in Figure 4, the H3 motif is hypothesized to be the ancestral motif since it is present in all three cryptic species studied here as well as the reference Australia species. However, the H1 motif is common to MEAM1, MED, and NW suggesting it either arose independently in each species or was found in the ancestor of the three species and gave rise to the H4 motif in NW and the H2 motif in the common ancestor of MEAM1 and MED. Untangling the phylogenetic history and demonstrating the phylogenetic utility of this compound microsatellite will require sequencing alleles from additional members of the cryptic species complex.

### Homoplasy and cryptic species

Mutations occurring in either the flanking region or the repeat region can lead to detectable size homoplasy in tandem repeat markers [57,58]. In addition, back mutations can lead to undetectable size homoplasy e.g. a 210 allele can arise either from a 203 allele or a 217 allele and distinguishing among these alternatives is impossible without parentage. The false signal given by apparently shared, but size homoplasious alleles in microsatellites is not generally considered a problem when many loci with high variability are used in population genetics [59], but see Balloux et al. [60]. Homoplasy can overestimate the frequency of shared alleles among species [51] and this has the potential to be misleading when those species are cryptic [61]. In this study, homoplasious alleles would have lead to an overestimate of alleles shared and hybridization among cryptic species if not sequenced and if not used in combination with mitochondrial DNA sequences. After sequencing, no evidence of interspecific hybridization was found at this locus.

Relative to the number of mitochondrial haplotypes found, very few *B. tabaci* haplotypes have spread globally [42]. Low genetic diversity is also apparent at the *Bem6* nuclear locus in North American samples of invasive MEAM1 and MED. This locus initially appeared diagnostic for the cryptic species MEAM1 and MED. Even after genotyping several thousand samples, 99% of MEAM1 whiteflies had the 216 and/or 223 alleles and 98% of MED samples had the 203 and/or 210 alleles (Table 2). But rare alleles and putative hybrids surfaced as sample size increased and, without sequencing, would have lead to misinterpretation of allele sharing among both native and invasive cryptic species and an overestimation of hybrid frequency. These pitfalls might be even greater in the native range of MEAM1 and MED due to higher genetic diversity. Researchers should therefore use caution when using microsatellites for diagnostic purposes.

## Conclusions

Sequencing 11 alleles at the putatively dinucleotide *Bem6* locus from three members of a cryptic species complex revealed four different heptanucleotide tandem repeat motifs. The sequencing data is consistent with step-wise evolution, suggesting the locus evolves like a microsatellite rather than a minisatellite. In addition, the alleles described may also have arisen via length independent slippage expected when copy number is low. In two of the species, a compound heptanucleotide repeat is formed and, to our knowledge, this is the first such report.

Homoplasious alleles at the *Bem6* locus would have lead to an overestimate of alleles shared and hybridization among cryptic species if not sequenced. After sequencing, no evidence of interspecific hybridization remained. These results highlight the need for caution when using microsatellites for cryptic species discrimination and diagnostics.

## Competing interest

Authors declare that they have no competing interests and are responsible for the content of this paper.

## Authors’ contributions

AMD conceived and conducted experiments, analyzed data, and drafted the manuscript. PMH conceived and conducted experiments and analyzed data. RGS conceived experiments, analyzed data, and edited the manuscript. CLM initiated and conceived experiments. All authors read and approved the manuscript.

## References

[B1] Sager R Ishida MR Chloroplast DNA in *Chlamydomonas* Proc Natl Acad Sci USA 1963 50 4 725 730 10.1073/pnas.50.4.725 14077504 PMC221252

[B2] Britten RJ Kohne DE Repeated sequences in DNA Science 1968 161 529 540 10.1126/science.161.3841.529 4874239

[B3] Tautz D Chakraborty R, Epplen JT, Jeffreys AJ Notes on the definition and nomenclature of tandemly repetitive DNA sequences DNA Fingerprinting: State of the Science, Volume 67 1993 Basel, Switzerland: Birkhauser Verlag 21 28 10.1007/978-3-0348-8583-6_2 8400689

[B4] Dean C Schmidt R Plant genomes: a current molecular description Annu Rev Plant Biol 1995 46 1 395 418 10.1146/annurev.pp.46.060195.002143

[B5] Navajas-Perez R Paterson AH Patterns of tandem repetition in plant whole genome assemblies Mol Genet Genomics 2009 281 6 579 590 10.1007/s00438-009-0433-y 19242726

[B6] Tamaki K Rapley R, Whitehouse D Minisatellite and microsatellite DNA typing analysis Molecular forensics 2007 Chichester, U.K: John Wiley & Sons 71 89

[B7] Fondon JW III Hammock EAD Hannan AJ King DG Simple sequence repeats: genetic modulators of brain function and behavior Trends Neurosci 2008 31 7 328 334 10.1016/j.tins.2008.03.006 18550185

[B8] Nakamura Y Leppert M O’Connell P Wolff R Holm T Culver M Martin C Fujimoto E Hoff M Kumlin E Variable number of tandem repeat (VNTR) markers for human gene mapping Science 1987 235 4796 1616 1622 10.1126/science.3029872 3029872

[B9] Tautz D Renz M Simple sequences are ubiquitous repetitive components of eukaryotic genomes Nucleic Acids Res 1984 12 10 4127 4138 10.1093/nar/12.10.4127 6328411 PMC318821

[B10] Litt M Luty JA A hypervariable microsatellite revealed by in vitro amplification of a dinucleotide repeat within the cardiac muscle actin gene Am J Hum Genet 1989 44 3 397 401 2563634 PMC1715430

[B11] Jeffreys AJ Wilson V Thein SL Hypervariable ‘minisatellite’ regions in human DNA Nature 1985 314 67 73 10.1038/314067a0 3856104

[B12] Weber JL Wong C Mutation of human short tandem repeats Hum Mol Genet 1993 2 8 1123 1128 10.1093/hmg/2.8.1123 8401493

[B13] Schug MD Mackay TFC Aquadro CF Low mutation rates of microsatellite loci in *Drosophila melanogaster* Nat Genet 1997 15 1 99 102 10.1038/ng0197-99 8988178

[B14] Baer CF Miyamoto MM Denver DR Mutation rate variation in multicellular eukaryotes: causes and consequences Nat Rev Genet 2007 8 619 631 17637734 10.1038/nrg2158

[B15] Levinson G Gutman GA Slipped-strand mispairing: a major mechanism for DNA sequence evolution Mol Biol Evol 1987 4 3 203 221 3328815 10.1093/oxfordjournals.molbev.a040442

[B16] Ellegren H Microsatellites: simple sequences with complex evolution Nat Rev Genet 2004 5 6 435 445 15153996 10.1038/nrg1348

[B17] Jeffreys AJ Neil DL Neumann R Repeat instability at human minisatellites arising from meiotic recombination EMBO J 1998 17 14 4147 4157 10.1093/emboj/17.14.4147 9670029 PMC1170747

[B18] Richard GF Paques F Mini-and microsatellite expansions: the recombination connection EMBO Rep 2000 1 2 122 126 10.1093/embo-reports/kvd031 11265750 PMC1084263

[B19] Brinkmann B Klintschar M Neuhuber F Hohne J Rolf B Mutation rate in human microsatellites: influence of the structure and length of the tandem repeat Am J Hum Genet 1998 62 6 1408 1415 10.1086/301869 9585597 PMC1377148

[B20] Kelkar YD Tyekucheva S Chiaromonte F Makova KD The genome-wide determinants of human and chimpanzee microsatellite evolution Genome Res 2008 18 1 30 38 18032720 10.1101/gr.7113408 PMC2134767

[B21] Primmer CR Saino N Muller AP Ellegren H others Directional evolution in germline microsatellite mutations Nat Genet 1996 13 4 391 10.1038/ng0896-391 8696329

[B22] Buschiazzo E Gemmell NJ The rise, fall and renaissance of microsatellites in eukaryotic genomes Bioessays 2006 28 10 1040 1050 10.1002/bies.20470 16998838

[B23] Huang QY Xu FH Shen H Deng HY Liu YJ Liu YZ Li JL Recker RR Deng HW Mutation patterns at dinucleotide microsatellite loci in humans Am J Hum Genet 2002 70 3 625 634 10.1086/338997 11793300 PMC384942

[B24] Nauta MJ Weissing FJ Constraints on allele size at microsatellite loci: implications for genetic differentiation Genetics 1996 143 2 1021 1032 8725247 10.1093/genetics/143.2.1021 PMC1207320

[B25] Grimaldi MC Crouau-Roy B Microsatellite allelic homoplasy due to variable flanking sequences J Mol Evol 1997 44 3 336 340 10.1007/PL00006151 9060400

[B26] Peakall R Gilmore S Keys W Morgante M Rafalski A Cross-species amplification of soybean (Glycine max) simple sequence repeats (SSRs) within the genus and other legume genera: implications for the transferability of SSRs in plants Mol Biol Evol 1998 15 10 1275 1287 10.1093/oxfordjournals.molbev.a025856 9787434

[B27] De Barro PJ Liu SS Boykin LM Dinsdale AB *Bemisia tabaci*: a statement of species status Annu Rev Entomol 2011 56 1 19 10.1146/annurev-ento-112408-085504 20690829

[B28] Dinsdale A Cook L Riginos C Buckley YM De Barro P Dinsdale A Refined global analysis of *Bemisia tabaci* (Hemiptera: Sternorrhyncha: Aleyrodoidea: Aleyrodidae) mitochondrial cytochrome oxidase 1 to identify species level genetic boundaries Ann Entomol Soc Am 2010 103 196 208 10.1603/AN09061

[B29] Perring TM The *Bemisia tabaci* species complex Crop Prot 2001 20 725 737 10.1016/S0261-2194(01)00109-0

[B30] Dennehy TJ Decain BA Harpold VS Zaborac M Morin S Fabrick JA Nichols RL Brown JK Byrne FJ Xianchun LI Extraordinary resistance to insecticides reveals exotic Q biotype of *Bemisia tabaci* in the New World J Econ Entomol 2010 103 2174 2185 10.1603/EC10239 21309242

[B31] McKenzie CL Hodges G Osborne LS Byrne FJ Shatters RG Jr Distribution of *Bemisia tabaci* (Hemiptera: Aleyrodidae) biotypes in Florida-investigating the Q invasion J Econ Entomol 2009 102 2 670 676 10.1603/029.102.0227 19449648

[B32] Elbaz M Lahav N Morin S Elbaz M Evidence for pre-zygotic reproductive barrier between the B and Q biotypes of *Bemisia tabaci* (Hemiptera: Aleyrodidae) Bull Entomol Res 2010 100 581 590 10.1017/S0007485309990630 20158928

[B33] Sun DB Xu J Luan JB Liu SS Reproductive incompatibility between the B and Q biotypes of the whitefly *Bemisia tabaci* in China: genetic and behavioral evidence Bull Entomol Res 2011 101 2 211 220 10.1017/S0007485310000416 21034521

[B34] McKenzie CL Bethke J Byrne FJ Chamberlin J Dennehy TJ Dickey AM Gilrein D Hall P Luswig S Oetting R Distribution of *Bemisia tabaci* (Hemiptera: Aleyrodidae) biotypes in North America after the Q invasion J Econ Entomol 2012 105 3 753 766 10.1603/EC11337 22812110

[B35] Dalton R Whitefly infestations: the Christmas invasion Nature 2006 443 898 900 10.1038/443898a 17066003

[B36] Shatters RG Jr Powell CA Boykin LM Liansheng H McKenzie CL Improved DNA barcoding method for Bemisia tabaci and related Aleyrodidae: development of universal and Bemisia tabaci biotype-specific mitochondrial cytochrome c oxidase I polymerase chain reaction primers J Econ Entomol 2009 102 2 750 758 10.1603/029.102.0236 19449657

[B37] Boykin LM Shatters RG Jr Rosell RC McKenzie CL Bagnall R De Barro P Frohlich DR Global relationships of *Bemisia tabaci* (Hemiptera: Aleyrodidae) revealed using bayesian analysis of mitochondrial COI DNA sequences Mol Phylogenet Evol 2007 44 1306 1319 10.1016/j.ympev.2007.04.020 17627853

[B38] De Barro PJ Scott KD Graham GC Lange CL Schutze MK Isolation and characterization of microsatellite loci in *Bemisia tabaci* Mol Ecol Notes 2003 3 40 43

[B39] Selkoe KA Toonen RJ Microsatellites for ecologists: a practical guide to using and evaluating microsatellite markers Ecol Lett 2006 9 5 615 629 10.1111/j.1461-0248.2006.00889.x 16643306

[B40] Maddison WP Maddison DR Mesquite: a modular system for evolutionary analysis 2001 In. 2.74 http://mesquiteproject.org edn

[B41] Tsagkarakou A Tsigenopoulos CS Gorman K Lagnel J Bedford ID Biotype status and genetic polymorphism of the whitefly Bemisia tabaci (Hemiptera: Aleyrodidae) in Greece: mitochondrial DNA and microsatellites Bull Entomol Res 2007 97 29 40 10.1017/S000748530700466X 17298679

[B42] De Barro P Ahmed MZ Genetic networking of the *Bemisia tabaci* cryptic species complex reveals pattern of biological invasions PLoS ONE 2011 6 10 e25579 10.1371/journal.pone.0025579 21998669 PMC3184991

[B43] Dickey AM Osborne LS Shatters RG Jr Hall PM McKenzie CL Population genetics of invasive *Bemisia tabaci* (Hemiptera: Aleyrodidae) cryptic species in the United States based on microsatellite markers J Econ Entomol 2013 106 3 1355 1364 10.1603/EC12512 23865202

[B44] Alemandri V De Barro P Bajerman N Arguello Caro EB Dumon AD Mattio MF Rodriguez SM Truol G Species within the *Bemisia tabaci* (Hemiptera: Aleyrodidae) complex in soybean and bean crops in Argentina J Econ Entomol 2012 105 1 48 53 10.1603/EC11161 22420254

[B45] Boykin LM Armstrong KF Kubatko L De Barro P Species delimitation and global biosecurity Evol Bioinform 2012 8 1 37 2012 10.6026/97320630008001 PMC3256992 22267902

[B46] Hannan AJ Tandem repeat polymorphisms: modulators of disease susceptibility and candidates for missing heritability Trends Genet 2010 26 2 59 65 10.1016/j.tig.2009.11.008 20036436

[B47] Mun JH Kim DJ Choi HK Gish J Debelle F Mudge J Denny R Endre G Saurat O Dudez AM Distribution of microsatellites in the genome of *Medicago truncatula*: a resource of genetic markers that integrate genetic and physical maps Genetics 2006 172 4 2541 2555 16489220 10.1534/genetics.105.054791 PMC1456377

[B48] Valdes AM Slatkin M Freimer NB Allele frequencies at microsatellite loci: the stepwise mutation model revisited Genetics 1993 133 3 737 749 8454213 10.1093/genetics/133.3.737 PMC1205356

[B49] Dieringer D Schlotterer C Two distinct modes of microsatellite mutation processes: evidence from the complete genomic sequences of nine species Genome Res 2003 13 10 2242 2251 10.1101/gr.1416703 14525926 PMC403688

[B50] Zhu Y Strassmann JE Queller DC Insertions, substitutions, and the origin of microsatellites Genet Res 2000 76 3 227 236 10.1017/S001667230000478X 11204970

[B51] Angers B Bernatchez L Complex evolution of a salmonid microsatellite locus and its consequences in inferring allelic divergence from size information Mol Biol Evol 1997 14 3 230 238 10.1093/oxfordjournals.molbev.a025759 9066791

[B52] Estoup A Tailliez C Cornuet JM Solignac M Size homoplasy and mutational processes of interrupted microsatellites in two bee species, *Apis mellifera* and *Bombus terrestris* (Apidae) Mol Biol Evol 1995 12 6 1074 1084 8524041 10.1093/oxfordjournals.molbev.a040282

[B53] Chistiakov DA Hellemans B Volckaert FAM Microsatellites and their genomic distribution, evolution, function, and applications: a review with special reference to fish genetics Aquaculture 2006 255 1 29 10.1016/j.aquaculture.2005.11.031

[B54] Dettman JR Taylor JW Mutation and evolution of microsatellite loci in neurospora Genetics 2004 168 1231 1248 10.1534/genetics.104.029322 15579682 PMC1448800

[B55] Kofler R Schlotterer C Luschutzky E Lelley T Survey of microsatellite clustering in eight fully sequenced species sheds light on the origin of compound microsatellites BMC Genomics 2008 9 612 10.1186/1471-2164-9-612 19091106 PMC2644718

[B56] Bacolla A Larson JE Collins JR Li J Milosavljevic A Stenson PD Cooper DN Wells RD Abundance and length of simple repeats in vertebrate genomes are determined by their structural properties Genome Res 2008 18 10 1545 1553 10.1101/gr.078303.108 18687880 PMC2556271

[B57] van Oppen MJH Rico C Turner GF Hewitt GM Extensive homoplasy, nonstepwise mutations, and shared ancestral polymorphism at a complex microsatellite locus in Lake Malawi cichlids Mol Biol Evol 2000 17 4 489 498 10.1093/oxfordjournals.molbev.a026329 10742041

[B58] Curtu AL Finkeldey R Gailing O Comparative sequencing of a microsatellite locus reveals size homoplasy within and between European oak species (Quercus spp.) Plant Mol Biol Rep 2004 22 4 339 346 10.1007/BF02772677

[B59] Estoup A Jarne P Cornuet JM Homoplasy and mutation model at microsatellite loci and their consequences for population genetics analysis Mol Ecol 2002 11 9 1591 1604 10.1046/j.1365-294X.2002.01576.x 12207711

[B60] Balloux F Brunner H Lugon-Moulin N Hausser J Goudet J Microsatellites can be misleading: an empirical and simulation study Evolution 2000 54 4 1414 1422 11005307 10.1111/j.0014-3820.2000.tb00573.x

[B61] Skrede I Borgen L Brochmann C Genetic structuring in three closely related circumpolar plant species: ALFP versus microsatellite markers and high-arctic versus arctic-alpine distributions Heredity 2009 102 293 302 10.1038/hdy.2008.120 19066622

